# Stable Population, Shifting Clades: A 17-Year Phylodynamic Study of IBV GI-19-like Strains in Spain Reveals the Relevance of Frequent Introduction Events, Local Dispersal and Recombination Events

**DOI:** 10.3390/v18010024

**Published:** 2025-12-23

**Authors:** Giovanni Franzo, Francesca Poletto, Matteo Legnardi, Riccardo Baston, Cristina Andolfatto, Laura Ramon, Marta Becerra, Mar Biarnés, Mattia Cecchinato, Claudia Maria Tucciarone

**Affiliations:** 1 Department of Animal Medicine, Production and Health (MAPS), Padua University, 35020 Legnaro, Italy; francesca.poletto.1@phd.unipd.it (F.P.); matteo.legnardi@unipd.it (M.L.); riccardo.baston@phd.unipd.it (R.B.); cristina.andolfatto.2@phd.unipd.it (C.A.); mattia.cecchinato@unipd.it (M.C.); claudiamaria.tucciarone@unipd.it (C.M.T.); 2 CESAC—Centre de Sanitat Avícola de Catalunya i Aragó, Avinguda de Castellvell, 32, 43206 Reus, Spain; lramon@cesac.org (L.R.); mbecerra@casac.org (M.B.); mbiarnes@cesac.net (M.B.)

**Keywords:** IBV, Spain, recombination, genotypes, epidemiology

## Abstract

Infectious bronchitis virus (IBV) remains a common pathogen in poultry production. Although its clinical and economic impact in Europe has markedly declined in recent decades due to extensive vaccination, ongoing viral circulation continues to pose risks to animal health and provides opportunities for viral evolution. In this study, we investigated the molecular epidemiology of GI-19 and related strains in Spain using samples collected between 2008 and 2025. Partial *S1* sequencing revealed a complex scenario involving three major clades and several minor ones, the latter likely resulting from independent introduction events from north-western Europe, particularly Denmark. Six distinct recombination events involving GI-13 and GI-19 parental strains—some apparently vaccine derived—were also identified, several of which showed wide geographical spread and long-term persistence. Both recombinant and non-recombinant variants were detected across multiple regions and production systems, indicating strong epidemiological connectivity among broilers, layers, and breeders. Although overall viral population size appeared stable over time, shifts in the predominance of specific clades and recombinant groups were observed, possibly reflecting fitness advantages of newly introduced or evolved variants and reduced cross-protection from existing immunity. These findings highlight the susceptibility of the poultry sector to repeated introductions, mixing, and the dissemination of IBV variants. Strengthened molecular surveillance and tailored control strategies, together with the periodic evaluation of vaccination practices and population immunity, are needed to limit viral circulation, reduce recombination opportunities, and mitigate the impact of IBV.

## 1. Introduction

Infectious bronchitis is a contagious disease caused by the infectious bronchitis virus (IBV), a member of the family *Coronaviridae*, genus *Gammacoronavirus*, species *Gammacoronavirus galli* (https://ictv.global/taxonomy, accessed on 5 September 2025). It is responsible for significant economic losses worldwide [1,2]. Most IBV strains cause respiratory disease and reproductive disorders, leading to a decline in productive performance. However, some nephropathogenic strains, or the superimposition of secondary bacterial infections, can result in severe mortality [3]. While prompt diagnosis and strict biosecurity measures can help reduce viral circulation and impact, the majority of control efforts currently rely on vaccination [4]. Live attenuated vaccines are more effective in inducing host immunity, including cellular and local one [5], and are therefore the only vaccines administered to short-lived birds. Despite their several advantages, some drawbacks are also associated with live vaccines, including potential residual virulence, the risk of reversion to virulence, and the possibility of recombination events [6,7]. Inactivated vaccines, although characterized by a higher safety profile, are generally less effective and typically used in long-living animal categories, such as layers and breeders, during the late stages of the production cycle, as a booster following priming with live vaccines to enhance humoral response and provide protection against reproductive disease [8].

However, the effectiveness of vaccination is undermined by IBV extensive genetic variability, which has emerged over time because of the high mutation and recombination rates shared by coronaviruses [9,10]. The heterogeneity of the spike (S) protein, particularly the S1 subunit, has been extensively investigated due to its biological role in mediating viral attachment to host cells, thus influencing cell and host tropism. For this reason, it also represents one of the main targets of the host immune response, particularly for neutralizing antibodies [11]. Its genetic variability has made the S1 subunit a key marker for molecular epidemiology, strain characterization, and classification [12]. Based on the phylogenetic analysis of this region, IBV has been classified into different genotypes, which are further divided into multiple lineages that may vary in terms of virulence and, more importantly, cross-protection [4]. In Europe, the GI-19 lineage has been representing the most relevant field variant, responsible for major productive losses [13]. Although its circulation appears to be decreasing thanks to the implementation of different control strategies, which also contribute to contain its clinical relevance [10], it still remains one of the main IBV lineages in several countries, including Spain.

Besides the development and use of homologous vaccines, the administration of vaccine combinations based on different lineages has proven to be a valuable approach to broaden the protection spectrum against heterologous challenges and is therefore widely adopted in the field [14,15]. However, in addition to vaccine strain selection, aspects such as proper administration strategy, planning, and coverage are equally important. Incorrect vaccine management can result in ineffective—or even detrimental—outcomes [8,16]. Both key factors—vaccine choice and application practices—can vary significantly between farms, companies, and even countries, potentially contributing to divergent epidemiological scenarios, even in neighbouring regions [17,18]. More generally, the overall organization of the poultry production system within a country can also directly or indirectly influence infection dynamics and disease epidemiology.

In spite of the relevance of poultry farming in Spain and the concerns raised by the ongoing circulation of IBV GI-19, no recent studies have been conducted to characterize the circulating strains, identify the underlying introduction and spreading patterns, describe their distribution and evolution, and ultimately re-evaluate the effectiveness of implemented control measures. Therefore, sequences collected from different Spanish regions were included in the study and analysed using bioinformatics and molecular epidemiology approaches, with the aim of filling this knowledge gap.

## 2. Materials and Methods

### 2.1. Spanish Sequences Dataset

Sequences included in the study originated from the diagnostic activity of the Centre de Sanitat Avícola de Catalunya i Aragó between 2011 and 2025 and were preliminary classified as GI-19 through BLAST v 2.17.0 analysis and comparison with reference strains.

All samples that tested positive using the generic IBV assay described by Callison et al. [19] were further characterized through amplification of the hypervariable region 3 (HVR3) of the *S1* gene using the XCE1 and XCE3 primers, as described by Cavanagh et al., 1999 [20]. Sanger sequencing was then performed in both directions using the same primers. Genotype and lineage classification was carried out by comparing the sequences with the reference strains provided by Valastro et al. [12], following alignment with MAFFT [21] and trimming to the genomic region sequenced in the present study. Sequences were preliminarily classified as GI-19 if they exhibited a genetic distance lower than 13% compared to the corresponding references. Information on collection region, farm or customer, host category, and sampling date were recorded for almost all included sequences. Additionally, Spanish sequences available in GenBank were downloaded and classified using a similar approach. To achieve a balance between sequence length and the number of relevant available sequences for which full coverage was present, the alignment was trimmed to a final length of 383 nt. When available, metadata such as collection country, date, and other fields matching those of the newly generated sequences were also annotated. The detection frequencies of the different clades within each production category were calculated together with their 95% confidence intervals using the Wilson method, implemented in *epitools* [22]. Statistical significance of differences in clade distribution across production categories was assessed using the Chi-squared test in R [23].

### 2.2. Recombination Analysis

All Spanish sequences plus the reference ones were aligned, the overlapping region was selected and analysed for recombination event and breakpoint identification using RDP4 [24]. Method-specific parameters were adjusted based on the characteristics of the dataset, following the recommendations in the RDP manual. An initial scan was performed using RDP, GENECONV, Chimaera, and 3Seq, while the complete set of available methods was employed in the subsequent refinement step. Only recombination events identified by at least three methods, with *p*-values < 10^−5^ and supported by Bonferroni correction, were considered reliable. During the preliminary analysis, several alternative criteria were also tested to demonstrate the consistency of the results across multiple settings. Parental strains and breakpoint identification were refined by performing manual bootscan analysis on the selected strains identified through the automated approach and all recombination events were graphically inspected to assess their reliability. Recombinant and non-recombinant strains were saved in different datasets.

### 2.3. Comparison with International Sequences

Non-recombinant sequences were compared with available GI-19 vaccine strains, and only those showing a percentage of identity lower than 99% were classified as field strains [25]. These field strains were then aligned with a dataset of international sequences downloaded from GenBank, selected based on the availability of collection country and date, and a genetic distance lower than 13% compared to the GI-19 reference sequences. A phylogenetic tree was reconstructed using the maximum likelihood approach implemented in IQ-TREE [26], selecting the substitution model with the lowest Akaike Information Criterion (AIC), as determined by the software. The robustness of the inferred topology was assessed using the Shimodaira–Hasegawa approximate likelihood ratio test (SH-aLRT) [27]. Monophyletic groups including more than 10 sequences were considered as clades and renamed accordingly.

### 2.4. Phylodynamic Analysis

The time to the most recent common ancestor (tMRCA), evolutionary rate, and viral population dynamics were reconstructed for all recombinant and non-recombinant clades that included more than 10 sequences, using the Bayesian serial coalescent framework implemented in BEAST X v10.5.0 [28]. The implemented Bayesian approach allowed for the simultaneous analysis of all datasets, with independent substitution models, molecular clocks, and genealogies (i.e., phylogenetic trees) for each clade. However, due to the limited number of sequences per clade, a hierarchical phylogenetic model was applied, in which the substitution model, gamma-distributed rate heterogeneity among sites, and molecular clock were linked across partitions [29]. This strategy allowed the sharing of evolutionary information across datasets while still accounting for some degree of heterogeneity between partitions. Essentially, this enabled information pooling to improve the precision of estimates within individual partitions. For all datasets, the HKY + Γ (gamma) substitution model combined with a relaxed molecular clock was used [30]. To reconstruct viral population dynamics in Spain, the Bayesian Skygrid model was selected [31]. Analyses were run for 200 million generations, sampling parameters and trees every 20,000 generations. Log files were analyzed in Tracer v1.7 [32]. Only results with effective sample size (ESS) values greater than 200 (after removing 20% burn-in) and adequate convergence and mixing were accepted. Parameter estimates were summarized as posterior means and 95% highest posterior density (HPD) intervals. Maximum clade credibility (MCC) trees were generated and annotated using TreeAnnotator v10.5.0 (part of the BEAST package).

## 3. Results

### 3.1. Spanish GI-19 Clades

A total of 164 GI-19-like sequences were generated (Acc. numbers PX442807-PX442970) in the present study and combined with an additional 32 sequences retrieved from GenBank (Acc. numbers GQ253482-GQ253486; KU934152-KU934178), covering the period from 2008 to 2025. Of these, 94 sequences were considered non-recombinant. Although six sequences showed high similarity to the L1148-based vaccine strain, all of them were sampled between 2008 and 2009, with one additional case in 2012—all occurring prior to the commercial registration of the corresponding vaccine. Therefore, these sequences were considered actual field strains.

The field strains could be grouped into three main clades (Supplementary Figure S1), comprising a total of 79 strains, while the remaining sequences were scattered throughout the phylogenetic tree as single branches or minor groups.

Among the countries showing the highest genetic similarity with the Spanish isolates, the Netherlands was the most represented, although related strains from Ukraine and China were also identified, typically collected within a five-year span of the Spanish cases. Each of the three main clades occupied a distinct position within the phylogenetic tree. Clade 1 included 11 Spanish sequences, sampled between 2019 and 2024. These strains were most closely related to isolates from the Netherlands and Ukraine (sampled in 2012) and again from The Netherlands (2020). An additional strain—PX442920—although not strictly part of Clade 1, showed a close relationship with these strains.

Clade 2 included 29 strains sampled between 2023 and 2025. These strains showed the closest genetic relationship with Dutch sequences. Finally, Clade 3 comprised 39 sequences collected between 2011 and 2015, which were closely related to strains sampled in the Netherlands, Germany, and Poland, collected during the same period.

### 3.2. Recombinant Clusters

**Figure 1:**
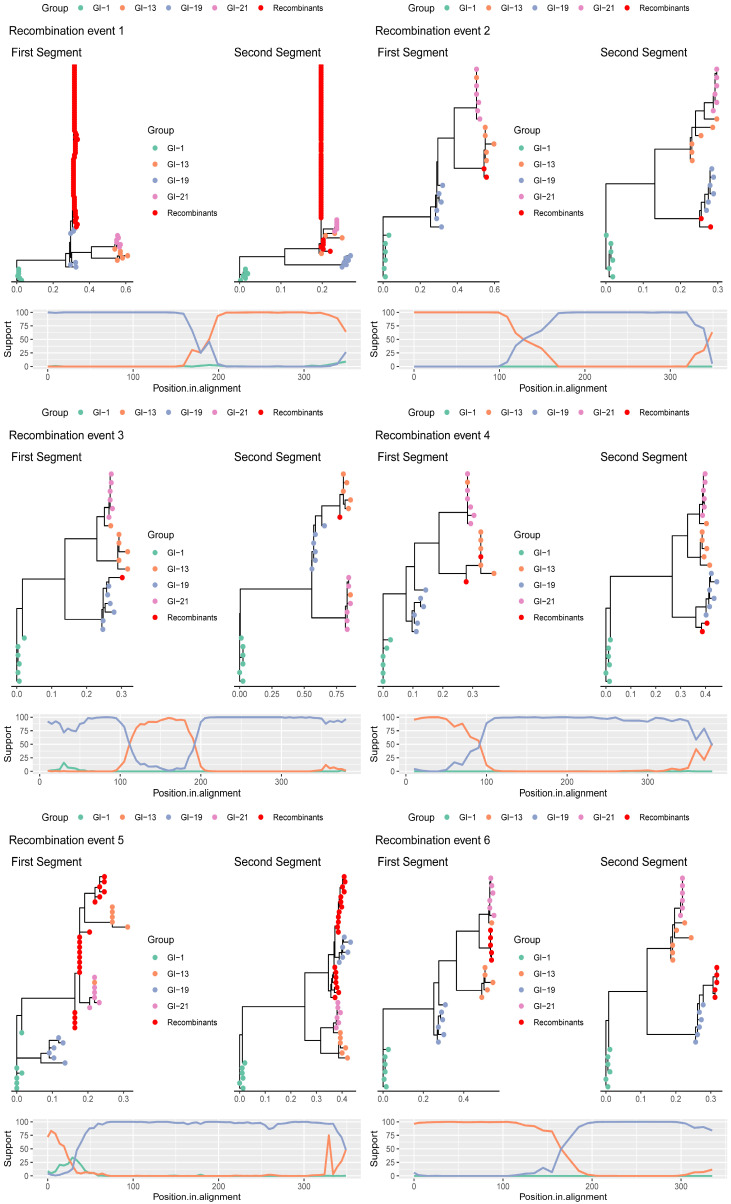
Depiction of the recombination events detected through RDP4. For each event, the phylogenetic trees reconstructed from the two parental regions are shown in the upper panels, while the lower panels report the results of the bootscan analysis. Recombinant and reference strains are color-coded. The analysis was performed on a ~380 nt region of the HVR3.

Six independent inter-lineage recombinant events were identified (Figure 1), listed and named according to the RDP confidence score. More in detail, recombination event 1 (Rec1) included 73 strains sampled between 2012 and 2014. Twenty-six of those sequences originated from another study and had been previously submitted to GenBank [33]. The recombination breakpoint was estimated approximately at position 180 of the considered alignment. The first segment parental strains belonged to GI-19, while the second was related to GI-13.

Recombination event 2 (Rec2) involved 2 strains sampled in 2020, having as minor and major parental strains members of the GI-13 and GI-19 lineages, respectively, with an estimated breakpoint at about position 130.

Recombination event 3 (Rec3), affecting one strain sampled in 2021 from a layer flock, involved a major GI-19 parental strain and a minor GI-13, providing an insert between position 113 and 192.

Recombination event 4 (Rec4) involved 2 strains also sampled from layer flock in 2024. The first segment originated from a GI-13 parental strain, while the second part from a GI-19.

Recombination event 5 (Rec5) involved 19 strains sampled between 2018 and 2025 from layers and one broiler breeder flock. The breakpoint was inferred at position 75. The phylogenetic trees generated for the two segments showed a certain heterogeneity, with two apparent subclades. However, the short segments involved, especially before the breakpoint, limited the resolution in phylogenetic tree and strain classification.

Finally, recombination event 6 (Rec6) involved five strains sampled in 2023–2024 from layers. The inferred breakpoint was at position 200, and the parental strains belonged to the GI-21 and GI-19 lineages, although in the first segment a reference strain formally classified as GI-13 clustered with GI-21, complicating the classification. The comparison of the recombinant strains with reference vaccines performed on the two sides of the detected breakpoint revealed only 3 potential vaccine parental strains (p-distance < 1%): a 4/91 (GI-13) for Rec1, 1/96 (GI-13) and D388 (GI-19) for Rec2 and D388 (GI-19) for Rec6.

### 3.3. Geographical and Host Distribution

**Figure 2:**
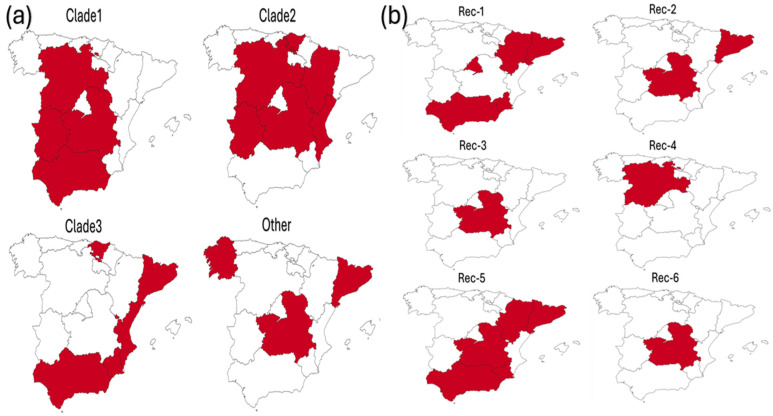
Maps of Spain highlighting in red the geographical distribution of the non-recombinant clades (**a**) and recombinant ones (**b**).

All major clades showed a broad geographical distribution, with Clade 1 tending to localize in the western–central part of Spain, Clade 2 in the central zone, and Clade 3 in the south, except for the Basque Country. In contrast, non-recombinant strains outside the main clades exhibited a more scattered distribution (Figure 2a).

**Figure 3:**
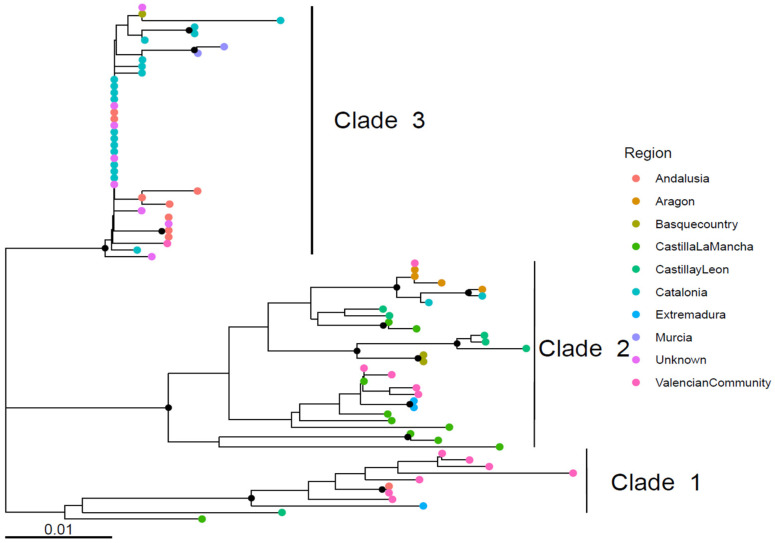
Phylogenetic tree of the three main non-recombinant clades. Collection regions are color-coded. Nodes with bootstrap support greater than 70% are indicated by solid black dots. The analysis was performed on a ~380 nt region of the HVR3.

Within each clade, phylogenetic analysis revealed no significant geographical clustering, with genetically divergent strains occurring within the same region and identical strains detected in different, often non-adjacent, regions (Figure 3).

A fully overlapping pattern was observed for recombinant strains, particularly for the most relevant groups—Rec1 and Rec5—which exhibited a longer history and a widespread geographical distribution (Figure 2b).

**Figure 4:**
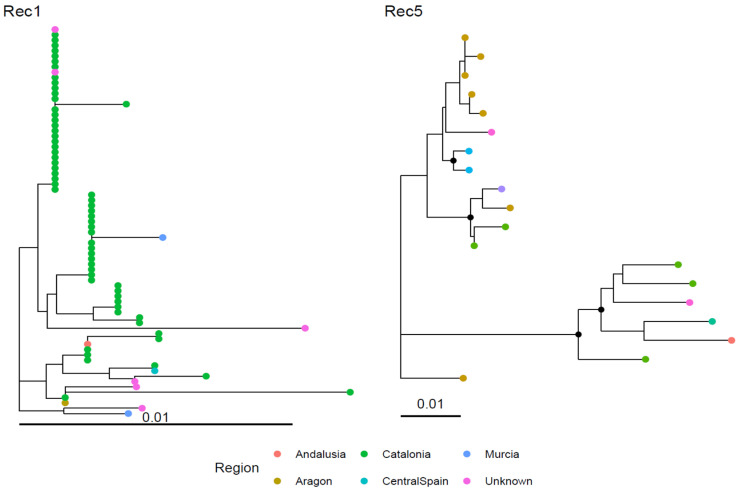
Phylogenetic tree of the two main recombinant clades. Collection regions are color-coded. Nodes with bootstrap support greater than 70% are indicated by solid black dots. The analysis was performed on a ~380 nt region of the HVR3.

As with the major clades, no clear geographical clustering was evident, with genetically identical strains more often originating from the same region but also occurring in geographically unrelated areas, and genetically divergent strains sometimes being sampled within the same region (Figure 4).

Considering the host distribution, Clade 1 was most detected in layers (10/11; 90.9%, 95% CI: 62.3–98.4) and only once in broilers (1/11; 9.1%, 95% CI: 1.6–37.7). Comparably, Clade 2 was detected only in long-living birds, mainly in layers (23/29; 79.3%, 95% CI: 61.6–90.2) and in broiler breeders (6/29; 20.7%, 95% CI: 9.8–38.4). In contrast, Clade 3 was detected mainly in broilers (29/39; 74.4%, 95% CI: 58.9–85.4) and to a lesser extent in layers (5/39; 12.8%, 95% CI: 5.6–26.8) and slow-growing chickens (3/39; 7.7%, 95% CI: 2.6–20.3), while for two strains no host information was available. Among recombinant strains, in Rec1 a comparable detection frequency was observed in broilers (38/73; 52.1%, 95% CI: 40.6–63.4) and layers (28/73; 38.4%, 95% CI: 28.1–49.8), then broiler breeders (4/73; 5.5%, 95% CI: 2.1–13.5) and finally slow-growing birds (3/73; 4.1%, 95% CI: 1.4–11.4). For Rec5, all strains originated from layers (18/19; 94.7%, 95% CI: 74.0–99.3), except one from broiler breeders (1/19; 5.3%, 95% CI: 0.9–26.0). Other recombinant clades were detected in long living birds only (Table 1).

No significant association was found between productive category and belonging to a recombinant/non-recombinant clade (*p* = 0.66), although the association between specific clades and productive categories was significant (*p* = 0.011), with some clades being over- or under-represented in certain categories (Supplementary Figure S2).

**Figure 5:**
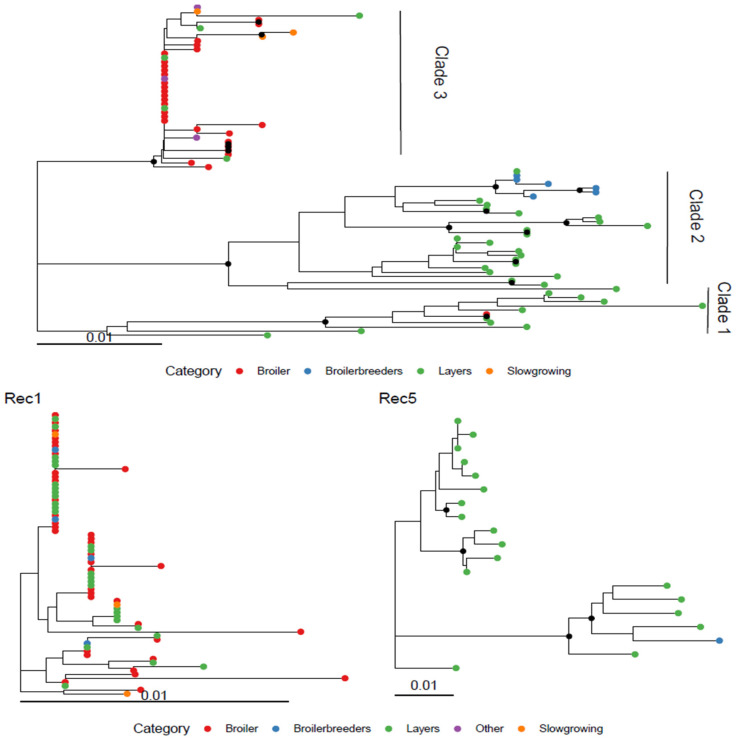
Phylogenetic tree of non-recombinant and the two main recombinant clades. Collection host categories are color-coded. Nodes with bootstrap support greater than 70% are indicated by solid black dots. The analysis was performed on a ~380 nt region of the HVR3.

The phylogenetic analysis revealed no productive category-based clustering, either among the non-recombinant clades or within Rec1 (Figure 5). In both cases, genetically related strains were collected across all host categories. For Rec5, the limited host variability prevented any firm conclusion; nevertheless, the strain derived from broiler breeders clustered together with those from layers (Figure 5).

### 3.4. Phylodynamic Analysis and Dynamics of Major Spanish Non Recombinant and Recombinant Clades

**Figure 6:**
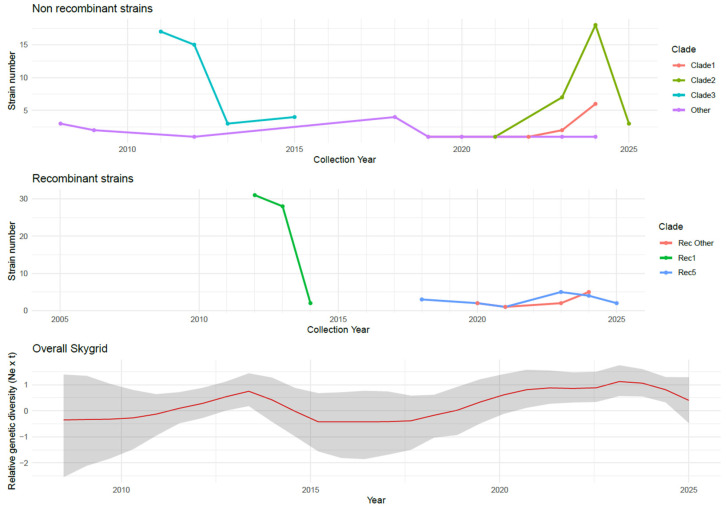
Temporal distribution of the non-recombinant clades (**upper panel**) and recombinant clades (**middle panel**). Different clades are color-coded. The (**lower panel**) shows the reconstruction of the overall IBV population dynamics, with the mean estimate depicted as a red line and the 95% HPD interval represented by the shaded area.

The distribution of GI-19 non-recombinant strains was characterized by the presence of two main clades, whose abundance peaked at different time points. Clade 3 was detected primarily between 2011 and 2015, while Clade 2 emerged from 2021 onward and remained prevalent through the end of the study period. Between 2022 and 2024, the emergence and increasing detection of Clade 1 was observed. In parallel, an approximately constant number of unclassified (out-of-clade) strains was detected from 2008 throughout the entire study period (Figure 6). The estimated tMRCA was in 2009.72 [95HPD: 2007.86–2010.91], 2018.58 [95HPD: 2016.36–2020.05] and 2017 [95HPD: 2015.98–2018.45] for Clade 3, 2 and 1, respectively.

A similar pattern was observed for the recombinant clusters (Figure 6), with all Rec1 strains detected between 2013 and 2015, and a more persistent but lower-level circulation of Rec5 observed between 2018 and 2025. Sporadic emergence of other recombinant strains has occurred since 2020. The estimated tMRCA for the main recombinant clusters was 2010 (95% HPD: 2008.20–2011.81) for the Rec1 clade, and 2017.24 (95% HPD: 2016.09–2017.95) for Rec5.

Despite the variability in dominant clades, the overall reconstruction of viral population dynamics of GI-19 and derived recombinant strains in Spain showed a certain degree of stability. However, a population increase was observed from the tMRCA up to approximately 2013, followed by a declining trend over the subsequent few years, and eventually a rebound, which appears to be still ongoing (Figure 6).

## 4. Discussion

In Europe, the GI-19 lineage has represented the main IBV-related threat to poultry production for many years, although the progressive implementation and refinement of control strategies have markedly mitigated its impact [10]. Nevertheless, substantial differences persist among countries. A previous study highlighted distinct patterns in viral population dynamics between Italy and Spain, which could be at least partially attributed to differences in vaccination strategies and protocols. In Italy, IBV outbreaks are now sporadic, and the disease is largely under control, particularly since vaccination—either homologous or heterologous—has been consistently applied at the hatchery [15]. Accordingly, the monitoring activities reveal that most of the detected strains are vaccine viruses, and field GI-19 detections are exceptional reports. This systematic and uniform approach to vaccination is therefore likely a key factor in maintaining viral circulation at low levels.

Although most samples originated from monitoring activities in the absence of overt clinical signs, the situation in Spain appears less stable: GI-19 and GI-19-like strains are still commonly detected, suggesting that while vaccination and other control measures are effective at reducing disease severity, they are apparently less effective at limiting circulation of the infectious agent. The reconstruction of the overall dynamics of IBV field strains (or QX-like) further supports this evidence, suggesting a stable trend, and even an increase in recent years. Moreover, this apparently simple pattern conceals a much more intricate scenario. First, Spain is highly interconnected from an epidemiological perspective, with foreign strains showing a high likelihood of becoming established in the country. The introduction of new variants has largely contributed to shaping IBV molecular epidemiology, with the strongest links being to Western European countries, particularly the Netherlands, from which significant importation of live poultry occurs. (https://comtradeplus.un.org/TradeFlow accessed on 10 October 2025). However, the over-representation of sequences from The Netherlands in public datasets could have biased the analysis. Although some Spanish sequences genetically related to foreign strains appeared as singletons or formed minor clusters—suggestive of epidemiological dead ends—others demonstrated successful persistence and spread over time. This pattern is indicative of limited biosecurity and reduced effectiveness of current control measures, a finding supported by additional evidence from the study. The other hallmark of Spanish IBV is the high detection frequency of recombinant strains, with two recombination events achieving notable evolutionary success. In all instances, although the breakpoint positions varied, recombination involved strains belonging to the GI-13 and GI-19 genotypes, a combination commonly described by other authors [33,34,35]. While IBV is known to be highly prone to recombination [36,37,38,39,40], it is evident that favourable conditions must be present for these events to occur, leading to the co-infection of the same subjects with different strains. While the circulation of multiple field strains is possible, the widespread use of live attenuated vaccines might be an additional concern, as it could artificially increase the likelihood of co-infection. Both GI-13 and GI-19 vaccines have been developed and applied in Spain. Although with some variability among poultry farms, companies, and over time, the vaccination of broilers as well as layer and breeder chicks generally involves a combination of GI-1 and GI-13 administered by spray at the hatchery. In broilers, GI-19 has also been introduced as an alternative to GI-13, particularly in southern Spain. Pullets and hens at rearing facilities are also subject to additional inactivated and live attenuated vaccines, based on GI-1, GI-13, and GI-19 (and, more sporadically, GI-12), administered via drinking water. Live attenuated vaccines—GI-1 and GI-13 in particular—have been shown to replicate persistently in chickens, circulate within flocks for extended periods, and even spread between farms [16,41,42,43]. Such capability can be further exacerbated when vaccination coverage is low, as often occurs when the vaccine is administered via drinking water. This allows transmission from vaccinated to unvaccinated birds and increases the risk of both rolling reactions and recombination [4], potentially explaining why most recombinants were observed in long-living birds, where live vaccines from different lineages are administered multiple times under suboptimal conditions. The detection of recombinants in broiler flocks could be explained by subsequent dispersal, as supported by the extensive exchange of both recombinant and non-recombinant strains between short- and long-lived birds, as demonstrated by phylogenetic analysis. However, a direct role of broiler flocks in the occurrence of recombination events cannot be excluded. While GI-13 and -19 only seem to be involved instead of GI-1 is still obscure. A shorted persistence in field condition or spreading potential can be involved [42]. Alternatively, or in addition, structural incompatibilities may decrease viral fitness when such genotypes are involved, particularly in the presence of unfavourable recombination breakpoints. This likely also explains why only a subset of the detected recombination events became relevant at the epidemiological level, persisting and spreading for at least a certain period.

It must nevertheless be stressed that for three recombination events only (Rec1, Rec2 and Rec6) the parental strains could be classified as vaccine strains based on the set genetic distance threshold, i.e., 4/91-, 1/96- and D388-based vaccines. The tMRCA of the recombinant clusters for which a reliable estimation was possible, typically predated the first available sequence by some years. Considering the high evolutionary rate of IBV [44,45,46]—which might be expected to be even higher in newly emerged strains under stronger selective pressure aimed at fitness re-optimization—substantial evolution could have occurred during that period, potentially concealing the relationship with vaccine strains for other recombination events. Nevertheless, GI-19 field strains were certainly involved at least in certain instances, as Rec1 originated years before the introduction of homologous vaccines. This finding, while supporting the role of live attenuated vaccines as potential parental strains, also highlights the risk of co-infection with field viruses. As a side note, the higher number of Rec1 sequences detected compared to more recent recombinant clusters—whose vaccine origin cannot be excluded—could suggest greater virulence when field strains are involved. However, the obtained results are not sufficient to support this statement, as both low- and high-virulence strains have been reported to originate from recombination events involving 4/91 strains [35,47]. Further research is therefore needed to obtain conclusive evidence. Regardless of the final outcome, and considering that most recombination events are likely disadvantageous (and thus a much higher number of recombination events probably occurred compared to those detected), the present study reveals that the Spanish poultry production system is vulnerable to the introduction of multiple strains—whether field or vaccine-derived—within the same farm. After viral mixing and recombination occurred, the same critical factors facilitated their dispersal. Rec1 and Rec5 showed remarkable spreading capability, fully overlapping that of non-recombinant strains and affecting a high number of Spanish regions within a limited time span. The substantial, unconstrained viral dispersal was further confirmed by phylogenetic analysis, which revealed not only the presence of different clades, but also of different variants of the same clade within the same regions, as well as identical strains detected in multiple regions. Even more surprisingly, strong epidemiological links involved different productive categories, once again highlighting the presence of multiple contact points that endanger the Spanish poultry production system. The determinants of such contacts among production flows—typically managed independently—should therefore receive further investigation.

A certain association between specific clades and host categories was observed. Although contingent epidemiological factors provide a plausible explanation, specific adaptations and preferential tropisms cannot be excluded [48], as has been suggested for other lineages [49,50].

While IBV field strains—recombinant or not—have been a constant threat overall, when considered individually they showed significant fluctuations over time, appearing, rising, and subsequently declining. A comparable pattern has been reported for the GI-19 lineage in the Netherlands and attributed either to the continuous competitive displacement of variants driven by ongoing adaptive evolution, or to a succession of distinct introductions and extinctions of field strains in livestock [48]. In the Spanish scenario also, it can be speculated that the introduction or emergence of new variants with genetically and phenotypically distinct features might confer a competitive advantage, as they are less likely to be recognized by the host population’s immunity [51], although conclusive evidence cannot be provided [48]. Both heterologous and homologous vaccination are effective, when properly managed, in providing adequate protection against clinical disease [8]. However, such protection is not sterilizing, and infection, viral shedding, and transmission can still occur, with varying intensity depending on the challenge strain. This is particularly true for homologous vaccination, whose mode of action relies on the genetic relatedness to the targeted field variant. The periodic emergence of new antigenic variants or recombination events—especially those affecting the S1 region—can jeopardize its effectiveness [5]. Notably, given the diagnostic purpose for which the sequences were originally generated, the results of the present study are based on the HVR3 of the *S1* gene. Although highly informative, this region represents a limited portion of the genome, coding for a single domain of the spike protein. Consequently, additional genetic variability and more complex recombination patterns may have been missed.

While this constitutes a limitation of the present study and should be addressed in future investigations, it further highlights the marked variability and evolutionary potential of this virus, as well as the challenges this may pose for its effective control.

While the progressive rise in population immunity was likely pivotal in shaping IBV epidemiology, the higher losses induced by these new strains, alone or in the presence of other co-infections and predisposing factors, compared to endemic ones, might have prompted the implementation of stricter and more effective control measures, as evidenced by the intensity of diagnostic activity and the short lag between the tMRCA and the first strain detection.

## 5. Conclusions

Overall, the present study highlights the challenging epidemiological situation of IBV field strains in Spain. The periodic introduction of new strains from foreign countries, combined with their rapid spreading capability across regions and host categories, underscores the limited effectiveness of current control strategies. The wide viral circulation, coupled with the administration of live attenuated vaccines—likely under suboptimal conditions, especially in long-lived birds—has created favourable conditions for the emergence of high-fitness recombinant strains, which displayed behaviour and epidemiological patterns comparable to those of field viruses. The occurrence of several waves of newly introduced or generated variants not only complicates the epidemiological scenario but also represents a major challenge from an immunological perspective. Strict monitoring activities and the establishment of control plans tailored to the molecular epidemiology and specific characteristics of the farming system should be implemented on a broad geographical scale and shared among producers and stakeholders. The effectiveness of these measures should be regularly assessed, both at the facility level (e.g., vaccine coverage, viral replication, development of protective immunity) and at the regional level (e.g., systematic sampling and diagnostic surveillance), to enable an objective evaluation of the strategies applied and their refinement, when necessary. Such measures would help prevent the emergence of susceptible niches for viral expansion, ultimately limiting viral circulation and spread, and thereby counteracting its evolutionary potential.

## Figures and Tables

**Table 1:** Count of the samples belonging to different clades or recombinant clades classified based on the host category.

Category	Clade1	Clade2	Clade3	Other	Rec1	Rec2	Rec3	Rec4	Rec5	Rec6
Broiler	1	0	29	7	38	0	0	0	0	0
Broiler breeders	0	6	0	1	4	1	0	0	1	0
Layers	10	23	5	7	28	1	1	2	18	5
Slow growing chicken	0	0	3	0	3	0	0	0	0	0
Other	0	0	2	0	0	0	0	0	0	0

## Data Availability

All sequences generated in the present study were made available in GenBank under the Acc. numbers PX442807-PX442970.

## Supplementary Materials

The following supporting information can be downloaded at: https://www.mdpi.com/article/10.3390/v18010024/s1, Figure S1. Maximum likelihood phylogenetic tree depicting the topology of the GI-19 lineage based on partial S1 sequences. Branch lengths are drawn to scale, with the scale bar representing the number of nucleotide substitutions per site. The three main Spanish clades are highlighted in different colors (red: Clade 1, green: Clade 2, blue: Clade 3); Figure S2. Mosaic plot depicting the relationship between host category and detected clade. The area of each cell is proportional to the count size. Cells have been colour-coded and lines dotted based on standardized residuals (a standardized residual greater than 2 or lower than −2 is indicative of statistical significance).
