# Supplementary figures and images for: Stable Population, Shifting Clades: A 17-Year Phylodynamic Study of IBV GI-19-like Strains in Spain Reveals the Relevance of Frequent Introduction Events, Local Dispersal and Recombination Events
Source: Viruses. 2025 Dec 23;18(1):24. doi: 10.3390/v18010024 (PMC12846379; doi:10.3390/v18010024)

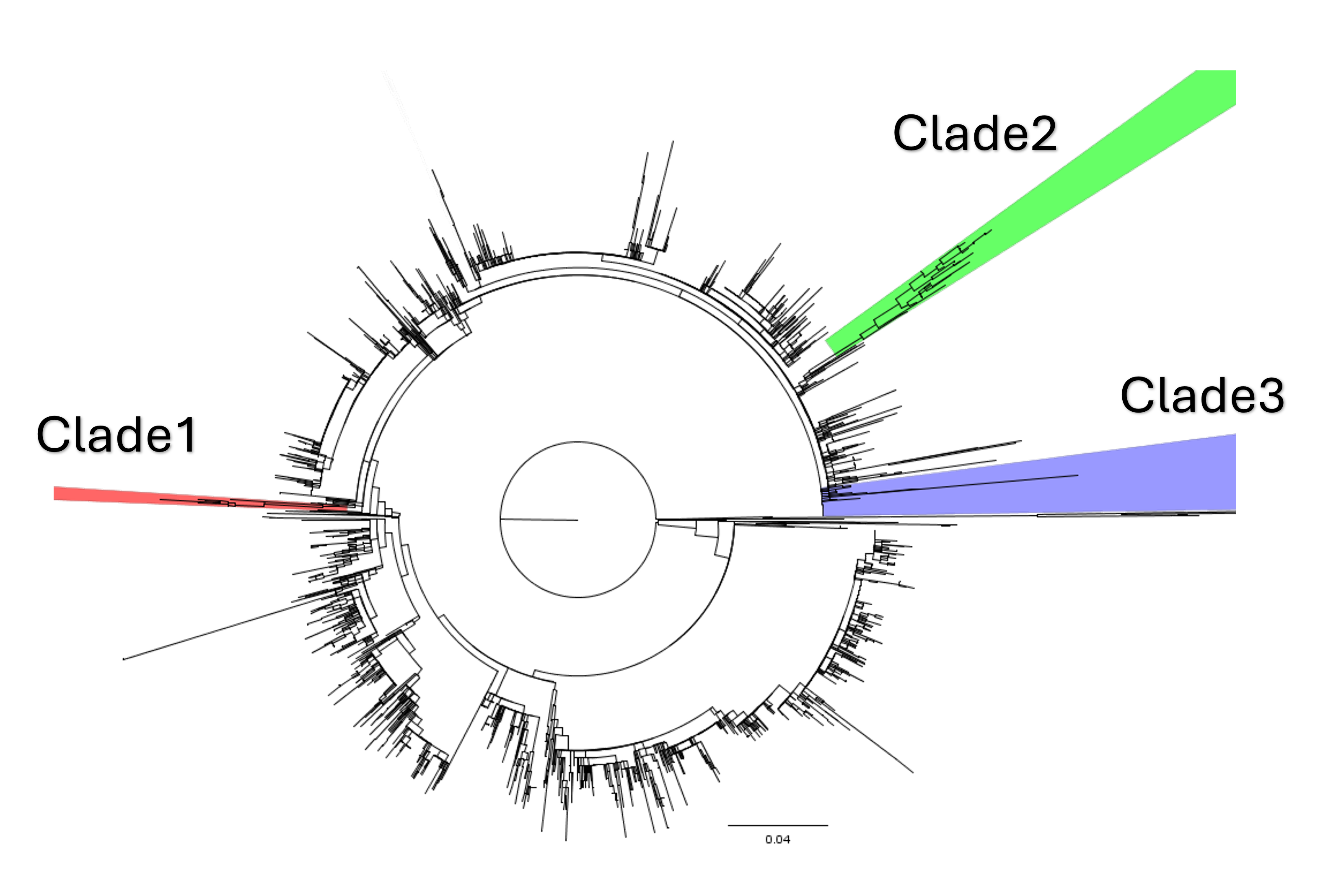

Supplement: Supplementary file 1 [file viruses-18-00024-s001.zip › Supplementary figure 1.png]

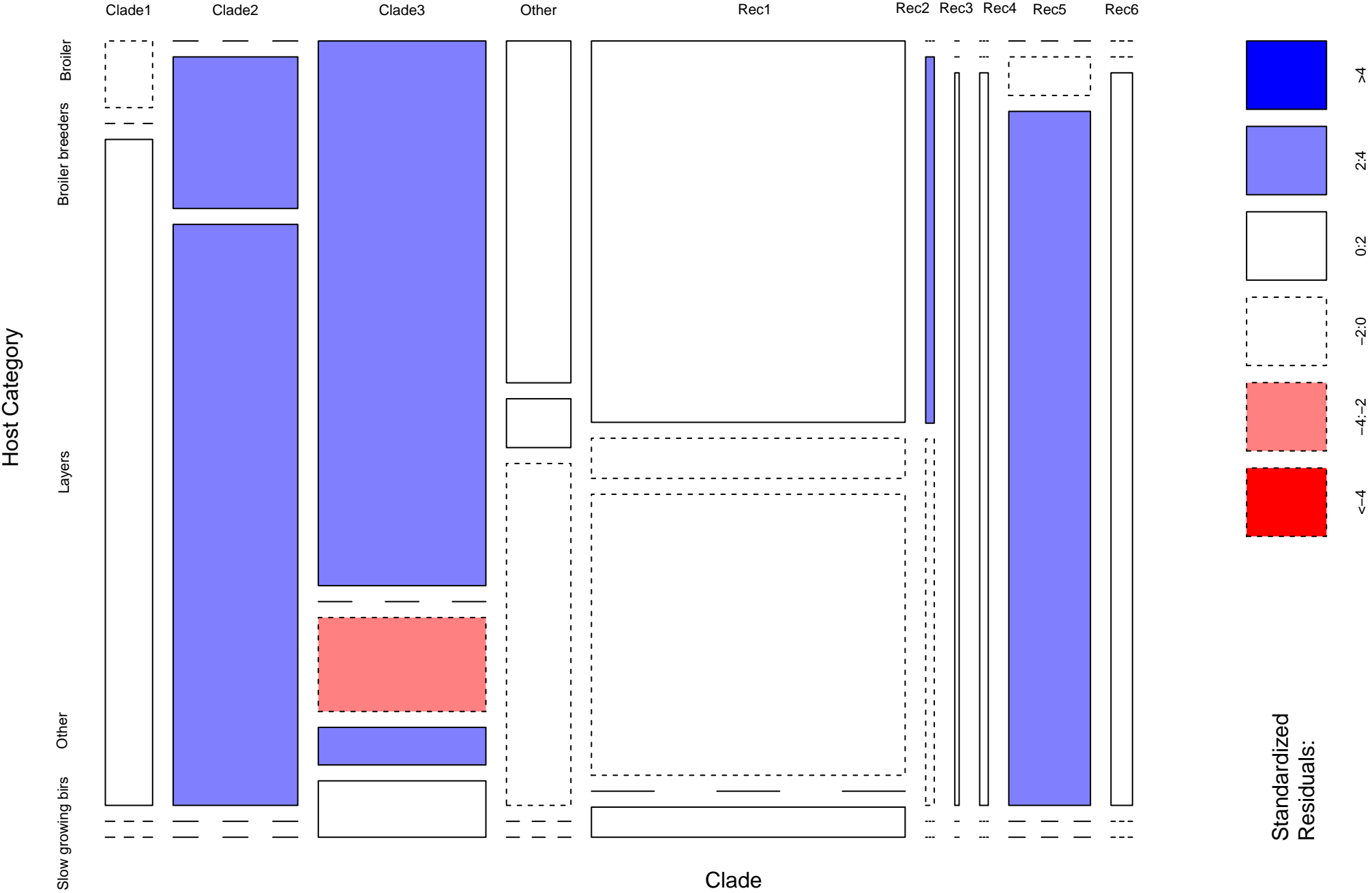

Supplement: Supplementary file 1 [file viruses-18-00024-s001.zip › Supplementary figure 2.pdf]
